# 

**Published:** 1890-04-12

**Authors:** 


					vol.
-April 12th, 1?9U.
ran
Monthly Farts, 6cL; post free 8d,
r?ered at thp r ^ ' x"uw* M\ ML JMk Jtt^ ? Monthly Parts, 6(L; post free 8fl.
?5^eneral Post Office as a Newspaper for Ks C-?* -?J \ i ? 0 , .
^"Ussion in the United Kingdom and Abroad. ^ Ditto per Annum 8s. post free.
0
9
%
Science, /Ifeetocine, IRumng anii jf^jilantljropg

				

